# Supplementary material for: A Case of Adaptive Laboratory Evolution (ALE): Biodegradation of Furfural by Pseudomonas pseudoalcaligenes CECT 5344
Source: Genes (Basel). 2019 Jun 29;10(7):499. doi: 10.3390/genes10070499 (PMC6678421; doi:10.3390/genes10070499)
Supplement: Supplementary file 1 [file genes-10-00499-s001.pdf]

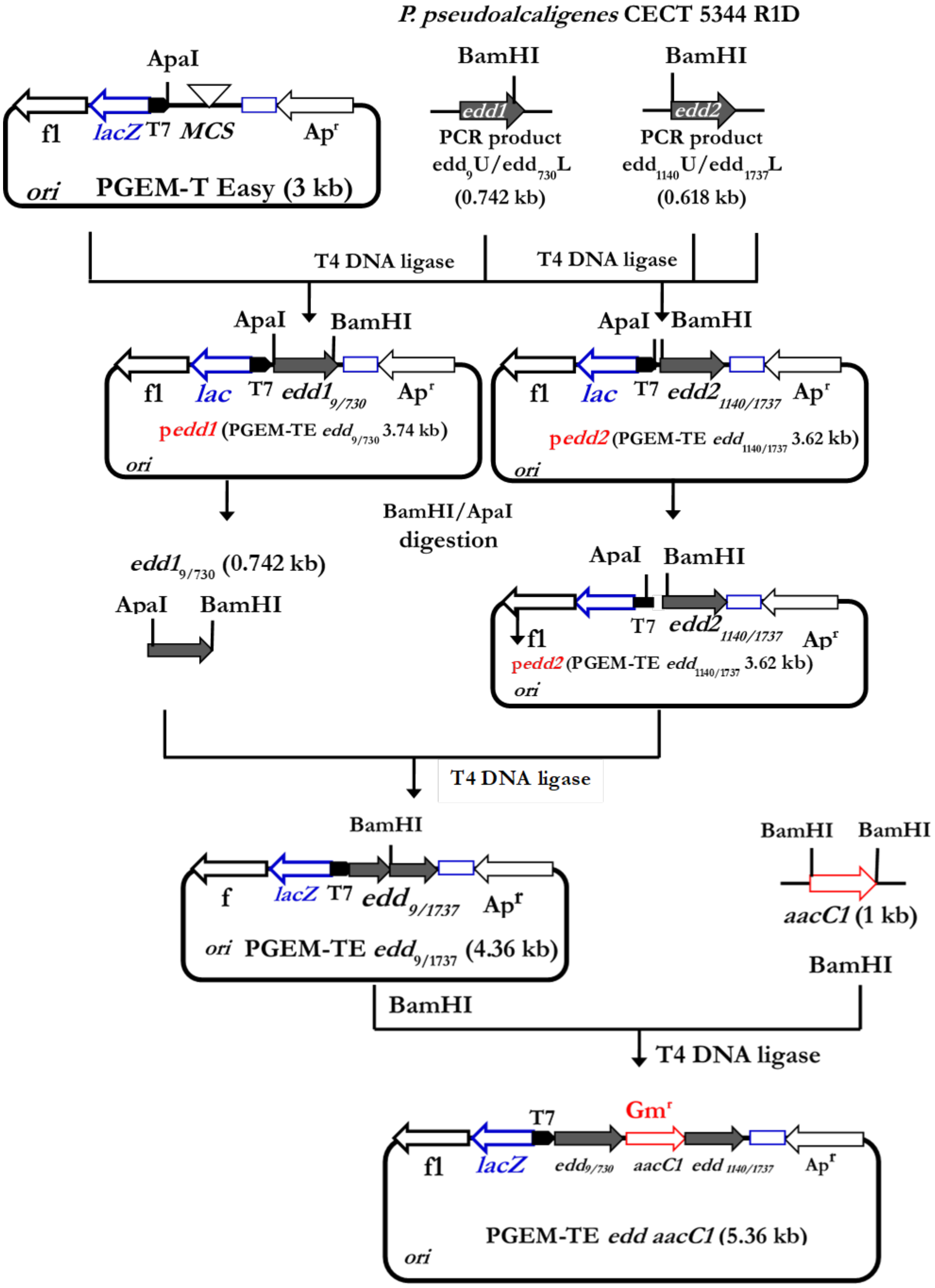

Figure S1. Schematic construction of PGEM-TE *edd aacC1* plasmid. T7: T7 RNA polymerase promoter; lacZ: β-galactosidase gene, f1: phage f1 region; ori: plasmid replication origin, MCS: Multi-Cloning Site. Two DNA fragments of the *edd* gene from *P. pseudoalcaligenes* CECT 5344 were obtained by PCR using specific primers and the genomic DNA of the bacterium as template. The gentamicine resistance gene (*aacC1*), amplified separately by PCR, was inserted in between them. The resulting plasmid, that is suicide in *P. pseudoalcaligenes*, was introduced by electroporation and the resulting Gm^r transformants analysed by PCR. A mutant in which the *edd* gene was interrupted by the Gm cassette by double recombination was selected for further analysis.

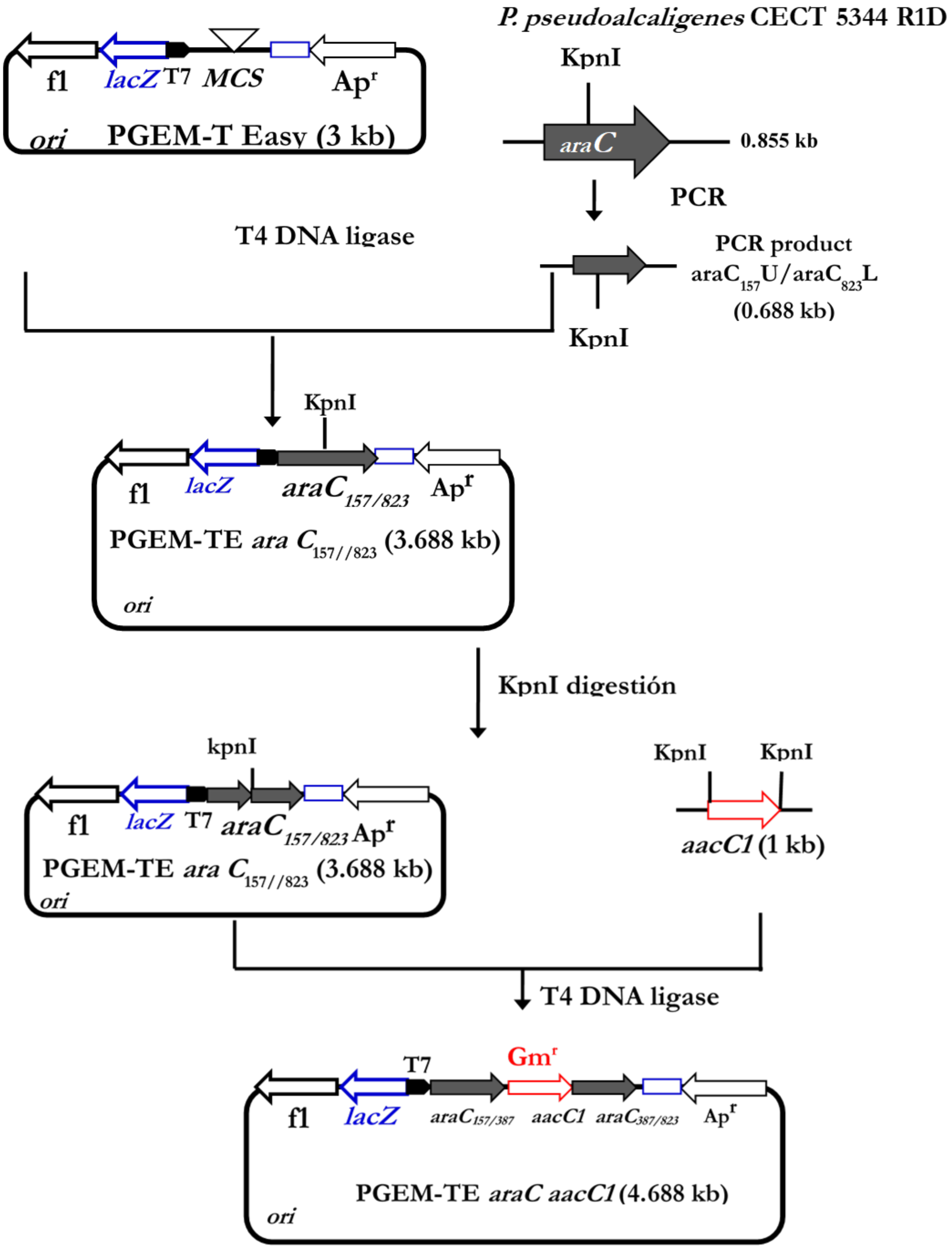

Figure S2. Schematic construction of PGEM-TE *araC aacC1* plasmid. T7: T7 RNA polymerase promoter; lacZ: β-galactosidase gene, f1: phage f1 region; ori: plasmid replication origin, MCS: Multi-Cloning Site. An internal DNA fragment of the *araC* gene from *P. pseudoalcaligenes* CECT 5344 was obtained by PCR using specific primers and the genomic DNA of the bacterium as template. The gentamicine resistance gene (*aacC1*), amplified separately by PCR, was inserted in a existing KpnI site. The resulting plasmid, that is suicide in *P. pseudoalcaligenes*, was introduced by electroporation and the resulting Gm^r transformants analysed by PCR. A mutant in which the *araC* gene was interrupted by the Gm cassette by double recombination was selected for further analysis.
